# Parietal bone osteoid osteoma: A rare cause of button sequestrum sign in pediatrics. Case report and review of literature

**DOI:** 10.1002/ccr3.6416

**Published:** 2022-10-11

**Authors:** Ali Mehri, Farrokh SeilanianToosi, Fariborz Samini, Javad Akhondian, Ali Reza Khooei, Narges Hashemi

**Affiliations:** ^1^ Endoscopic and Minimally Invasive Surgery Research Center Mashhad University of Medical Sciences Mashhad Iran; ^2^ Department of Radiology School of Medicine Mashhad University of Medical Sciences Mashhad Iran; ^3^ Department of Neurosurgery School of Medicine Mashhad University of Medical Sciences Mashhad Iran; ^4^ Department of Pediatrics School of Medicine Mashhad University of Medical Sciences Mashhad Iran; ^5^ Department of Pathology School of Medicine Mashhad University of Medical Sciences Mashhad Iran

**Keywords:** button sequestrum sign, osteoid osteoma, parietal bone, pediatrics

## Abstract

The current study evaluates a rare case of parietal bone osteoid osteoma in pediatrics and review the differential diagnosis of button sequestrum sign in the literature. A 12‐year‐old girl expressed localized pain in the right parietal bone. MRI represented enhancing nodule with button sequestrum sign appearance.

## Abstract 2

Due to the rarity of condition in children, osteoid osteoma should be considered as a differential diagnosis of button sequestrum sign in magnetic resonance imaging in children.

## 1 CASE PRESENTATION

A twelve‐year‐old girl was referred to Ghaem Hospital neurology center, complaining of chronic dull pain in the right side of the head. She estimated the pain as level seven out of 10, worsening by combing and relatively constant with no radiation. No history of previous head trauma was identified. Physical examination merely revealed moderate tenderness on the right parietal bone with no visible or palpable lesion or lump on her head or face. As well, no other significant pathologic finding including visual, sensory, or neurologic disturbances was identified. She had an appendectomy 1 month before the current presentation due to acute appendicitis, which was consequently followed by a generalized seizure the next day after surgery. Her Electroencephalography (EEG) depicted considerable abnormalities that necessitated treatment with Carbamazepine 200 mg twice a day (B.I.D) for the following next 9 months. Of note, no relevant family history of similar headache, seizure, or epilepsy was identified, and the seizure did not occur again. The lesion was intended to be removed entirely by performing a craniotomy. Bone cement was then used to accomplish a cranioplasty; a surgical intervention used to repair cranial defects for both cosmetic and functional purposes. She recovered without incident and was pain‐free. The histology findings supported the osteoid osteoma diagnosis.

## 2 IMAGE FINDINGS

**FIGURE 1:**
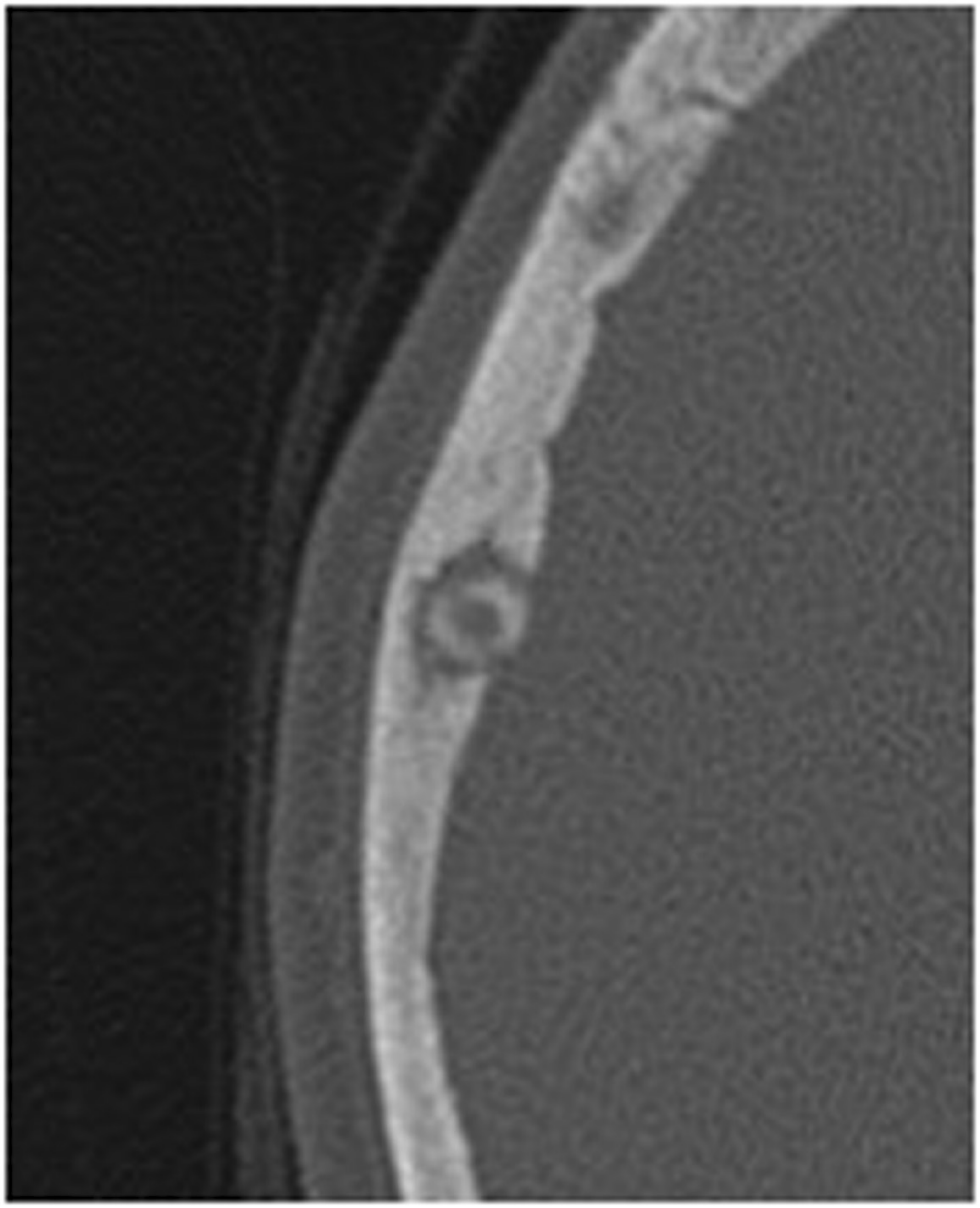
Axial bone window CT image shows a small rounded well‐defined lytic lesion surrounded by sclerosis and expansion of the diploic space in the right parietal area

**FIGURE 2:**
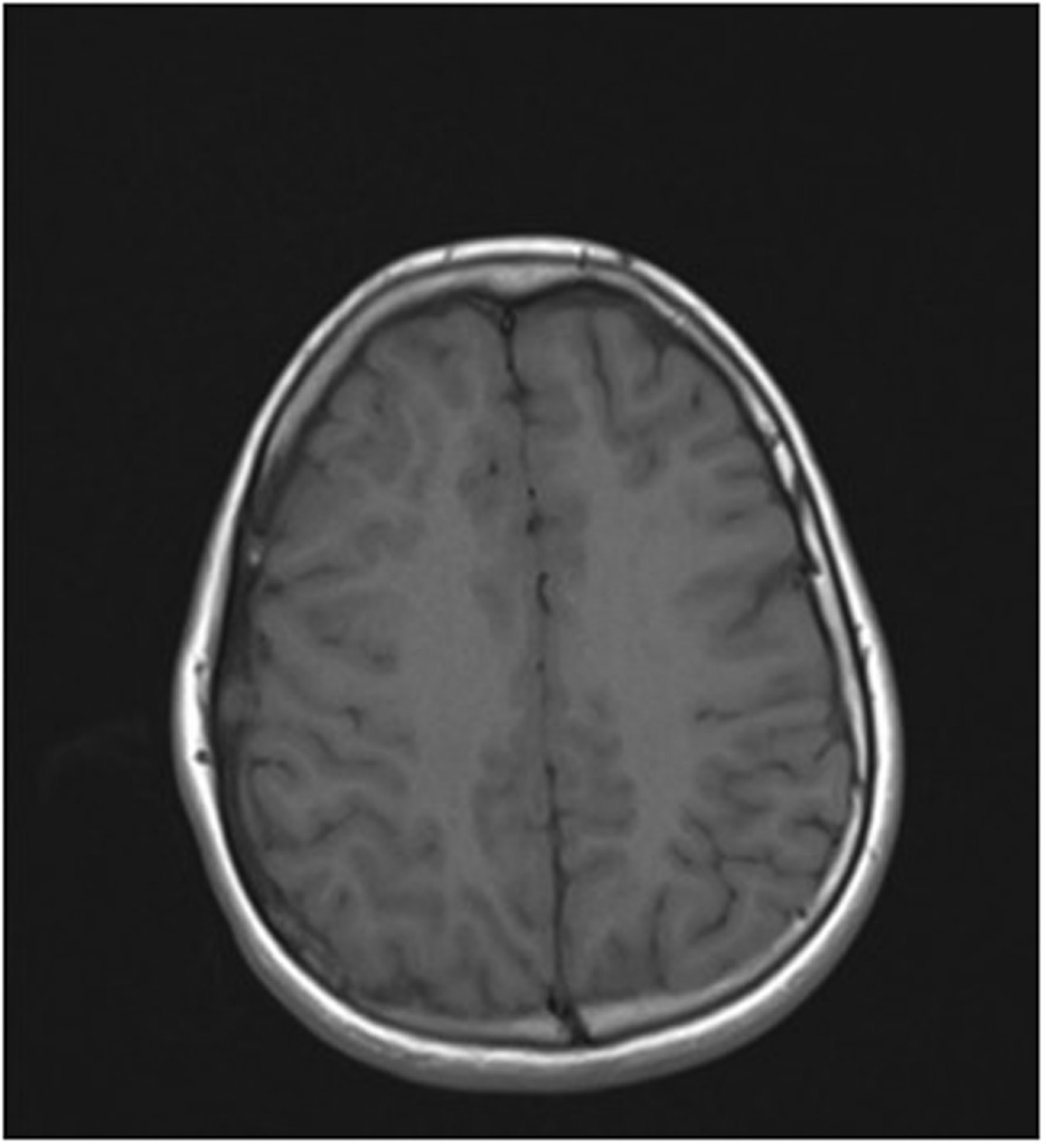
Axial T1‐weighted MR image shows an isointense lesion in the diploic space. Diploic space widening is depicted

**FIGURE 3:**
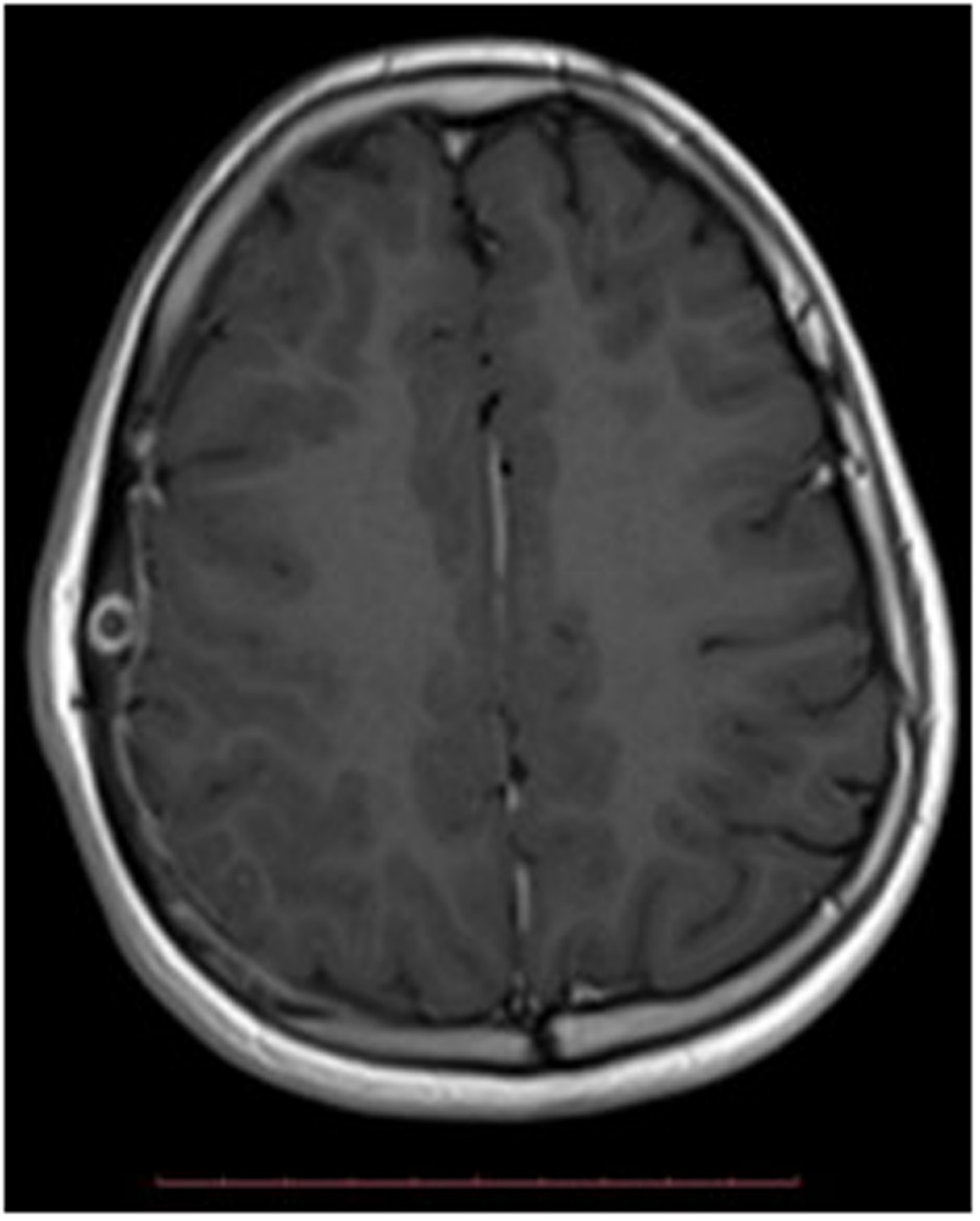
Axial T1‐weighted plus contrast MR image shows ring enhancement and also adjacent pachymeningeal enhancement

**FIGURE 4:**
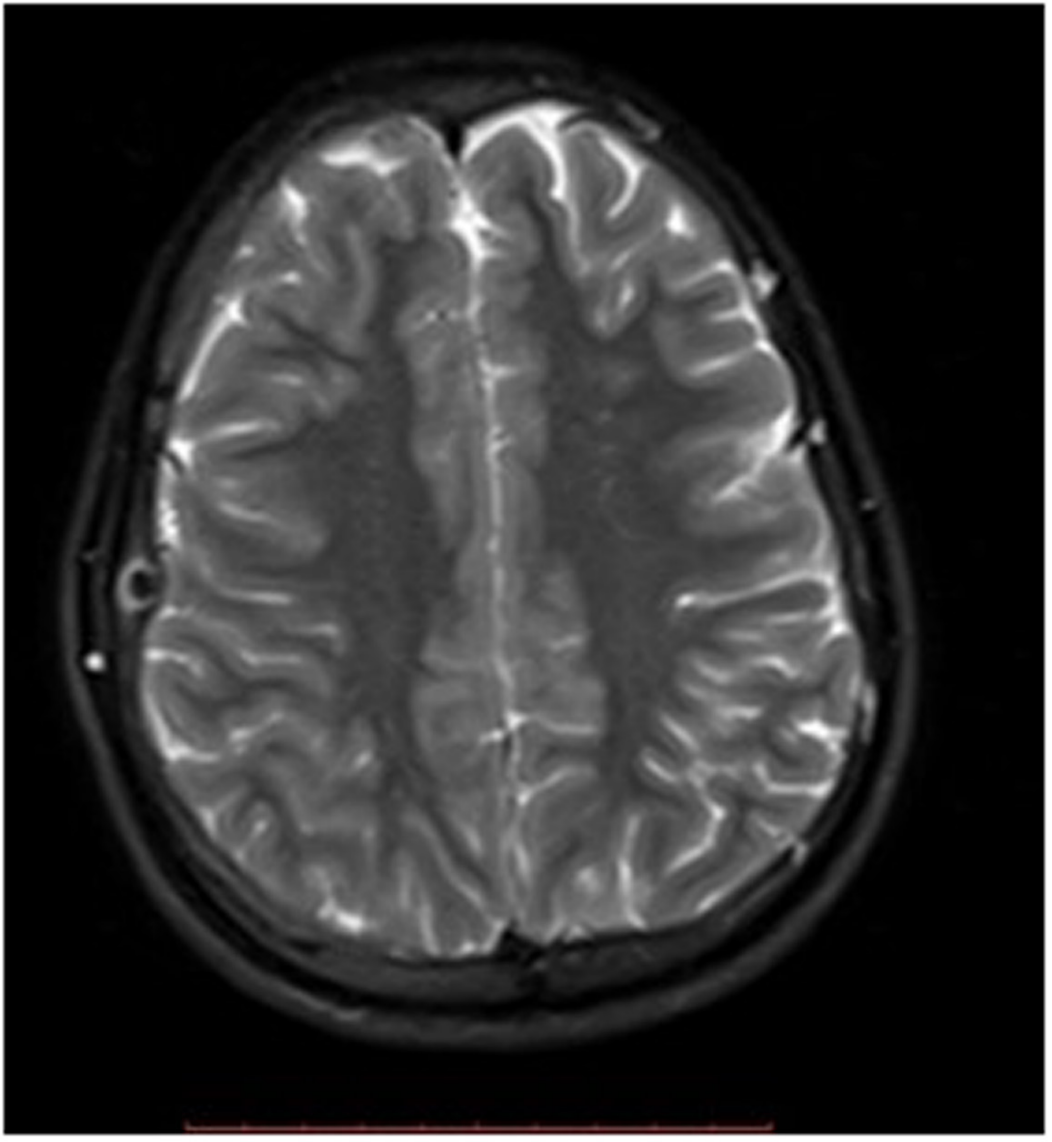
Axial T2‐weighted MR image shows a low signal intensity centrally with high signal intensity peripherally in the diploic space

**FIGURE 5:**
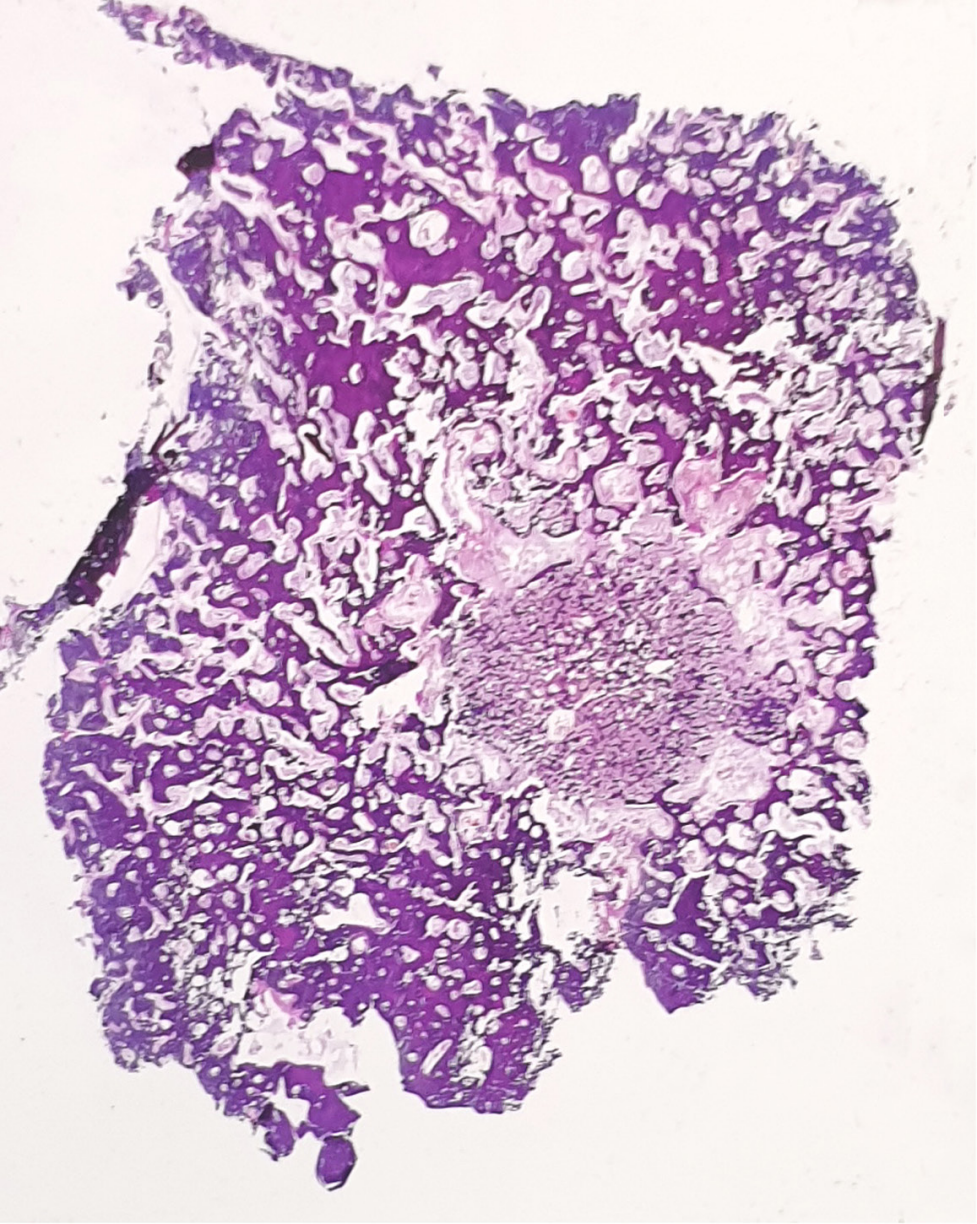
Whole mount appearance of the lesion shows a well‐defined nidus encompassed by sclerotic host bone, H&E stain

**FIGURE 6:**
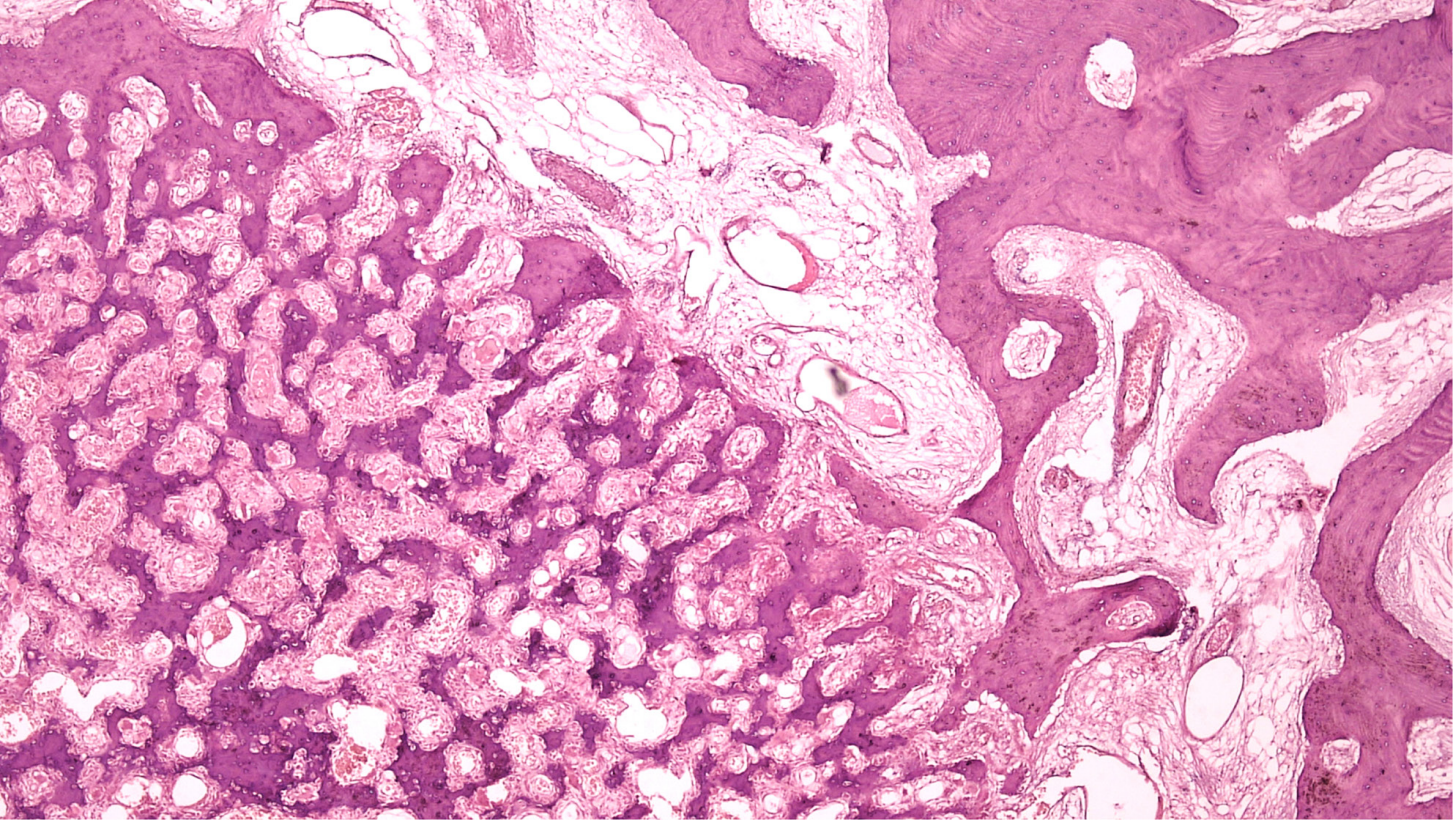
The nidus is well demarcated which is composing of a network of immature bony trabeculae surrounded by sclerotic host bony trabeculae, H&E stain(x40)

**FIGURE 7:**
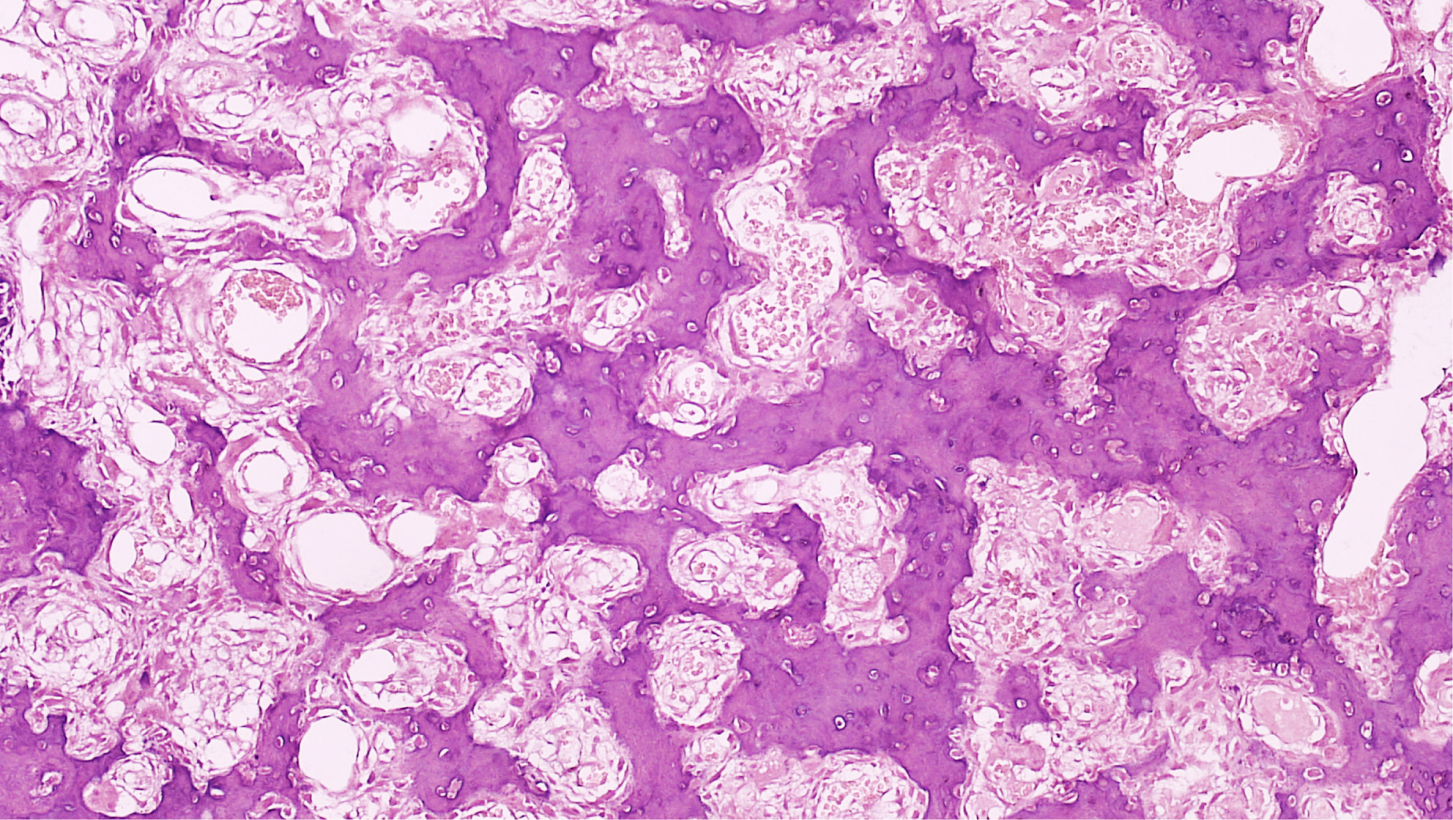
The Nidus is composed of interconnecting mineralized osteoid spicules embedding in a richly vascularized stroma, H&E stain(x100)

**FIGURE 8:**
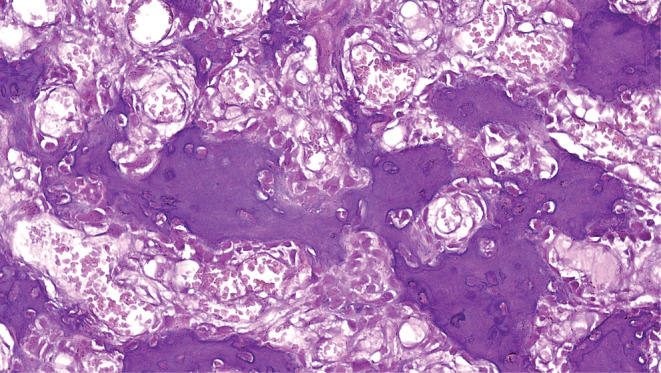
Osteoid spicules depict immature mineralization and are rimmed with benign plump osteoblasts; H&E stain(x400)

The Brain CT Scan and MRI (performed with and without contrast, in multi‐planar and different time echoes), demonstrated an enhancing nodule, 7 mm in diameter located in the right parietal bone (Figures 1, 2, 3, 4). Therefore, with the initial diagnosis of eosinophilic granuloma, the patient underwent surgery for ablation of the lesion. Histopathological examination revealed an intraosseous well‐defined nidus, consisting of anastomosing mineralized osteoid trabeculae rimmed with plump osteoblasts embedding in a vascular‐rich stroma, surrounded by sclerotic host bone, which was consistent with Osteoid Osteoma of bone (Figures 5, 6, 7, 8).

## 3 DISCUSSION

Osteoid osteoma is a benign bone‐forming tumor in children and adolescents, often presenting as a small round radiolucent nidus with a sclerotic margin in radiographic images. It is the cause of approximately 12% of benign bone tumors, usually occurring in the 2nd decade of life in the lower extremities, particularly in the proximal femur.^1^, ^2^ Moreover, it shows a strong male‐to‐female ratio, affecting boys 2–3 times more than girls.^3^ Patients describe increasing pain (especially at night) regardless of their daily activity. However, approximately 25% of cases cannot be diagnosed by radiographic patterns, necessitating other modalities like computerized tomography (CT) scan or magnetic resonance imaging (MRI).^4^ The Button Sequestrum Sign is a bony opacity in the center of a lucent area, which first was described by Wells in 1956 as a diagnostic view of the Eosinophilic Granuloma of bone. However, further studies revealed a similar appearance in the other bone lesions such as osteoid osteoma.^5^ Most of the time, it is found in radiography; however, a CT scan can be a further help in more controversial cases. MedLine database was checked by “Parietal Bone” [Mesh] and “Osteoma, Osteoid” [Mesh] search strategy. There were just two cases of parietal bone osteoid osteoma, which further emphasizes that the parietal bone is a rare localization for osteoid osteoma.^6^, ^7^ Several case reports have discussed the differential diagnosis of the button sequestrum sign. The summaries are categorized by disease and site of calcification for future research (Table 1).

**TABLE 1:** summary of several case reports about the differential diagnosis of button squestrum sign

	Disease	Articles	Location
1	Eosinophilic Granuloma	Wells/Sholkoff et al./Rosen et al./Helms et al.^8^, ^9^	Parietal/occipital/ occipital bone/Non defined
2	Metastatic Cancer	Rosen et al.^9^	Parietal bone
3	Tuberculous Osteitis	Rosen et al.^9^	Parietal bone
4	Meningioma	Sholkoff et al.^8^	Skull bone
5	Osteomyelitis	Rosen et al./Helms et al.^9^	Skull bone/Non defined
6	Dermoid Cyst	Sholkoff et al.^8^	Frontal bone
7	Radiation Necrosis	Rosen et al.^9^	Skull bone
8	Iatrogenic button sequestrum	Sholkoff et al.^8^	Skull bone
9	Multiple Staphylococcal Abscesses	Sholkoff et al.^8^	parietal bone
10	Fibrosarcoma	Helms et al.^10^	Non defined
11	Osteoid osteoma	Helms et al./Liu et al.^10^, ^11^	Non defined/Parietal & rib bones
12	Iatrogenic	Sholkoff et al.^8^	Skull bone

## 4 CONCLUSION

Due to the rarity of this condition in children, osteoid osteoma should be considered as a differential diagnosis of button sequestrum sign in magnetic resonance imaging. (1).

## AUTHOR CONTRIBUTION

Ali Mehri involved in writing the manuscript and submitting. Farrokh SeilanianToosi involved in describing the MRI and CT scan Images. Fariborz Samini involved in performing the surgery. Javad Akhondian and Narges Hashemi involved in patient care and manuscript proofing. Alireza Khooei involved in describing the histopathological samples.

## CONFLICT OF INTEREST

The authors declare that they have no conflict of interest.

## ETHICAL APPROVAL

Written informed consent was obtained from the patient's parent to publish this report in accordance with the journal's patient consent policy.

## CONSENT

Written informed consent was obtained from the patient to publish this report in accordance with the journal's patient consent policy.

## Data Availability

Data sharing not applicable to this article as no datasets were generated or analysed during the current study.
